# Fitness-Related Short Video Engagement and Body Appreciation: Gender-Differentiated Indirect Pathways via Exercise Identity and Mindful Eating

**DOI:** 10.3390/bs16091685

**Published:** 2026-09-18

**Authors:** Yuqiang Zhang, Xiaofei Xu

**Affiliations:** 1 Department of Physical Education, Anhui Agricultural University, Hefei 230036, China; zhyq8092@ahau.edu.cn; 2 Department of Psychology, University of Science and Technology of China, Hefei 230026, China

**Keywords:** fitness-related short-video, social media engagement, body appreciation, exercise identity, mindful eating, college students

## Abstract

This cross-sectional study examined the association between self-reported fitness-related short video social media engagement and body appreciation among Chinese college students, focusing on the mediating roles of exercise identity and mindful eating and whether these pathways differed by gender. A total of 536 Chinese college students (270 women, 266 men) completed self-report measures. Multigroup structural equation modeling (SEM) with 5000 bootstrap resamples was used to test the hypothesized mediation pathways and formally compare indirect effects across genders. Measurement invariance was examined; only body appreciation achieved partial metric invariance, and the exercise identity scale showed suboptimal configural fit, so gender comparisons involving the other latent variables should be interpreted with caution. The serial mediation pathway (engagement → exercise identity → mindful eating → body appreciation) was significant only among women (effect = 0.069, 95% CI [0.037, 0.111]), with a significant gender difference (Women–Men diff = 0.062, 95% CI [0.019, 0.111]). The indirect pathway through exercise identity alone (engagement → exercise identity → body appreciation) was significant only among men (effect = 0.124, 95% CI [0.059, 0.199]), also with a significant gender difference (Women–Men diff = −0.111, 95% CI [−0.204, −0.022]). These findings suggest that the processes linking fitness-related short-video engagement and body appreciation may differ by gender. Among women, the observed indirect association was consistent with a sequential pathway involving both exercise identity and mindful eating, whereas among men, the observed indirect association was primarily explained by exercise identity. These findings are correlational and hypothesis-generating and require replication in longitudinal and experimental research before any causal or interventional implications can be drawn.

## 1. Introduction

The proliferation of short-video social media platforms has fundamentally transformed how young adults access information, entertain themselves, and shape their lifestyles (Wang et al., 2023). In China, this trend is particularly pronounced: as of December 2025, the number of short-video users had reached 1.074 billion, accounting for 95.40% of all internet users (China Internet Network Information Center, 2026). Among the diverse content categories on these platforms, fitness-related short videos—featuring workout routines, athletic challenges, and “fitspiration” material—have become particularly popular among young audiences (Liu & Luo, 2023). Such content has been associated with viewers’ body image and health behaviors through processes such as social modeling and parasocial interaction (Fardouly et al., 2018; Tiggemann & Zaccardo, 2015).

Body appreciation—defined as accepting, maintaining favorable opinions of, and respecting one’s body—is a core component of positive body image (Tylka & Wood-Barcalow, 2015). A large body of empirical work has shown that social media engagement is associated with body image outcomes (Donovan et al., 2020; Rodgers et al., 2011). Most of this evidence has focused on fashion-, beauty-, or thin-ideal-related content (e.g., idealized celebrity images, cosmetic tutorials, and “thinspiration” posts), and findings consistently show associations between such engagement and adverse body image outcomes, including greater appearance comparison, body dissatisfaction, social appearance anxiety, restrictive eating, and disordered eating behaviors (Alleva & Tylka, 2021; Holland & Tiggemann, 2016; Martin et al., 2023; Prichard et al., 2020; Raggatt et al., 2018; Tiggemann & Zaccardo, 2015). By contrast, fitness-related content has only recently attracted sustained scholarly attention, and the emerging evidence is considerably more mixed. Some studies have found that fitness-related social media engagement may be associated with greater appearance comparison, body dissatisfaction, and restrictive eating, particularly when viewers interpret the content as another standard of bodily perfection to be attained (Martin et al., 2023; Prichard et al., 2020). Other studies, however, have reported beneficial associations with exercise participation, appreciation of body functionality, and more positive overall body image (Alleva & Tylka, 2021). Although content type may moderate these associations, the present study does not directly distinguish among fitness content subtypes; rather, it focuses on general self-reported engagement with fitness-related short videos.

These discrepant findings suggest that the associations between fitness-related short-video social media engagement and body image are not uniformly positive or negative. Prior work indicates that content characteristics may help explain these differences. Specifically, content emphasizing body functionality, health, and exercise competence has been linked to adaptive outcomes, including greater body appreciation and reduced appearance comparison (Alleva & Tylka, 2021; Vintró-Alcaraz et al., 2025). In contrast, appearance-oriented fitness content—often termed “fitspiration” or “fitspo”—emphasizes muscularity, leanness, and an idealized “fit” physique and shares substantial overlap with thinspiration content in its associations with appearance comparison, body dissatisfaction, and disordered-eating cognitions. Some studies have even reported comparable adverse effects on women’s mood, body image, and dietary restraint (Martin et al., 2023; Prichard et al., 2020; Tiggemann & Zaccardo, 2015, 2018; Yee et al., 2020).

Although prior research has documented associations between fitness-related social media engagement and body image outcomes. The underlying psychological processes—particularly in the short-video context—remain insufficiently understood. In the present study, fitness-related short-video engagement refers to self-reported attention to, information seeking about, and perceived relevance of fitness-related short-video content, rather than objective viewing time or cumulative dosage (hereinafter referred to as engagement). Moreover, whether these mediating pathways differ by gender remains an open question. Addressing these gaps is theoretically and practically important because it may help clarify previously overlooked gender-differentiated patterns and inform more targeted approaches to promoting positive body image among college students.

To help reconcile these inconsistent findings and provide a framework for interpreting the association between repeated engagement with fitness-related short videos and body appreciation, we draw on cultivation theory as the overarching framework for this study. Originally proposed by Gerbner to explain the cumulative effects of television on viewers’ perceptions of social reality, cultivation theory posits that greater immersion in a media environment is theorized to increases the likelihood that individuals will internalize its recurring themes and messages as reflections of the real world (Morgan & Shanahan, 2010; Steinfeld & Lev-On, 2020). Although initially developed for traditional broadcast media, the theory has since been extended to social media and short-video platforms, where algorithmic recommendations can create highly consistent and repetitive patterns of content engagement (Romann & Oeldorf-Hirsch, 2025). Cultivation theory provides a broad interpretive framework for understanding how repeated engagement with themed media content may be associated with self-relevant cognitions. However, because the present study does not directly measure cumulative exposure, viewing duration, or algorithmic dosage, cultivation theory is used here as an interpretive framework rather than as a formal dosage model to be tested.

Drawing on cultivation theory, we propose that repeated engagement may be associated with viewers’ self-relevant cognitions and behavioral orientations rather than functioning as a purely passive experience. Specifically, such engagement may be associated with stronger identification with the role of being an exerciser (i.e., exercise identity) and a nonjudgmental, awareness-based approach to eating (i.e., mindful eating), both of which are theorized to be positively associated with body appreciation. Accordingly, we hypothesize a positive association between engagement and body appreciation (H1). To examine whether the proposed mediation pathways differ by gender—an underexplored question—we use multigroup structural equation modeling to compare indirect effects across groups.

**Hypothesis** **1.**  *Engagement is positively associated with body appreciation among college students (total effect), with this association expected to be mediated by exercise identity and mindful eating.*

The first proposed mediator is exercise identity, defined as the extent to which an individual internalizes the role of being a person who exercises (Anderson & Cychosz, 1994). Exercise identity has been consistently associated with exercise behavior (Su et al., 2024; Verkooijen & Bruijn, 2013). Beyond behavior, exercise identity also has important cognitive and affective correlates. Research has shown that exercise-identity strength is positively associated with self-regulatory efficacy and social self-efficacy, which, in turn, are associated with more favorable body image outcomes (Strachan et al., 2009). These findings suggest that exercise identity may be associated with a cognitive pathway through which engagement may be associated with body appreciation. Importantly, this distinction matters because identity, rather than behavior alone, may represent one hypothesized pathway linking media engagement with self-perception and health-related cognitions (Anderson & Cychosz, 1994). This distinction is important because identity, rather than behavior alone, may be one process through which media content is associated with self-perception and subsequent health-related cognitions.

The second proposed mediator is mindful eating, defined as attending to food and eating experiences with nonjudgmental, present-moment awareness (Framson et al., 2009). Empirical evidence has linked mindful eating to several beneficial psychological outcomes, including less emotional and binge eating, lower body dissatisfaction, and greater body appreciation (Osborne et al., 2022; Silva et al., 2025; Webb et al., 2018). The association between exercise identity and mindful eating is theoretically plausible but remains relatively underexamined. Prior work has linked regular exercise habits and health-oriented lifestyles to greater mindful eating (Isiklar et al., 2025), and mindful eating has in turn been associated with more positive body image (Özyalçın & Yılmaz, 2025). However, these studies did not directly assess exercise identity as specified in the present model. More indirectly, recent evidence indicates that positive embodiment is positively associated with mindful eating and intuitive exercise, and that embodiment mediates the association between identity-related experiences and these mindfulness-based health practices (Pringle et al., 2026). Drawing on this rationale, we propose that a stronger exercise identity may be associated with greater body awareness and self-regulation, which in turn may be associated with more mindful eating. Because direct empirical evidence for this specific pathway is limited, we frame it as a theoretically plausible and relatively underexamined hypothesis rather than an established relationship.

However, the association between exercise identity- and eating-related attitudes is not uniformly adaptive. Some studies have cautioned that exercise identity—particularly when combined with media pressure to attain an idealized “fit” appearance—may be associated with greater body dissatisfaction and unhealthy eating behaviors (Palermo & Rancourt, 2021; L. Shi et al., 2024). These mixed findings further underscore the need to examine how exercise identity and mindful eating operate together. The present study focuses on the general strength of exercise identity rather than on appearance-driven or compulsive exercise identity; the latter may be associated with more negative outcomes (Palermo & Rancourt, 2021). Rather than functioning independently, exercise identity may be associated with a more functional, health-oriented self-perception, which may then relate to mindful eating and, ultimately, body appreciation. Integrating cultivation theory with the positive body image framework, we propose the following hypotheses:

**Hypothesis** **2.**  *Exercise identity mediates the association between engagement and body appreciation.*

**Hypothesis** **3.**  *Mindful eating mediates the association between engagement and body appreciation.*

**Hypothesis** **4.**  *Exercise identity and mindful eating sequentially mediate the association between engagement and body appreciation (i.e., engagement → exercise identity → mindful eating → body appreciation).*

## 2. Materials and Methods

### 2.1. Procedures and Participants

This study received an exempt-status determination from the Medical Research Ethics Committee of the First Affiliated Hospital of the University of Science and Technology of China (IRB approval number: 2026-ky274; see Supplementary File S1) on 1 June 2026. All co-authors completed relevant research ethics training and were involved in the study’s design and implementation, ensuring familiarity with relevant ethical procedures. The survey was administered from 2 June to 9 June 2026, and all responses collected during this period were included in the initial screening. A total of 560 participants were recruited through convenience sampling from 12 public elective course sections across three universities in Eastern China (Zhejiang Province), Central China (Anhui Province), and Western China (Sichuan Province), with section sizes ranging from 35 to 60 students. Given the geographic and cultural diversity of mainland China, this multi-region sampling strategy was intended to capture variation across regional subcultural contexts. The 12 public elective course sections were temporary teaching groups formed through voluntary course selection, with students coming from different majors and academic years. Although these sections were more heterogeneous than intact administrative classes, participants completed the survey within the same classroom sessions. We therefore acknowledge that some degree of within-section dependence could not be ruled out, and we evaluated its potential impact through a sensitivity analysis reported below. Section identifiers were not retained in the final dataset; therefore, cluster-adjusted inference could not be conducted.

Participants voluntarily provided digital informed consent before data collection. They completed the online questionnaire, hosted on Questionnaire Star platform (https://www.wjx.cn/, accessed on 10 June 2026), by scanning a quick-response (QR) code and could withdraw at any time without penalty. No incentives were offered. Instructors were present only to facilitate the orderly administration of the survey and did not monitor or review individual responses. To ensure full anonymity, no identifying information (e.g., student names, student numbers, or IP addresses) was collected. The study was presented as a survey of lifestyle behaviors, social media use, and body image, and participants were assured that their data would remain confidential.

Three pairs of validity-check items were included to identify invalid response patterns: (1) consistency checks involving height format, (2) verification of item completeness, and (3) checks for inattentive or random responding. Of the 560 questionnaires collected, 24 were excluded: 8 because of an incorrect height format, 5 because gender was not reported, and 11 because of missing items. No participants were excluded for inattentive or random responding. The final sample therefore comprised 536 participants (valid response rate = 95.71%; 270 women, 266 men). All participants were native Chinese speakers aged 18–26 years, with a mean age of 19.57 years (SD = 1.61). The majority of them self-identified as Han Chinese (93.66%). Mean BMI was 20.53 kg/m^2^ (SD = 3.18 kg/m^2^) for women and 22.49 kg/m^2^ (SD = 3.70 kg/m^2^) for men. Following conventional standards, an alpha level of 0.05 and a target power of 0.80 were adopted. However, we did not conduct a model-specific Monte Carlo power analysis for the present multigroup indirect-effect contrasts. This limitation is revisited in the Limitations section. The achieved sample size of 270 women and 266 men is comparable to sample sizes commonly used in multigroup SEM, but statistical power for detecting between-group differences in specific indirect effects depends on the sizes of all constituent paths, their covariances, measurement reliability, and the magnitude of the between-group difference. The present study may therefore have limited power to detect small between-group differences, and the absence of a model-specific power analysis should be considered when interpreting the indirect-effect contrasts.

### 2.2. Measures

#### 2.2.1. Fitness-Related Short-Video Social Media Engagement

The original scale was developed to assess fashion-related content (Yu & Zhang, 2025) and was adapted to fitness content by replacing references to fashion with references to fitness. The adapted version was reviewed by three experts in health psychology and pilot-tested with 60 college students to ensure clarity and relevance. The scale comprised 5 items: (1) I often follow fitness- or exercise-related content on short-video social media. (2) I actively search for fitness- or exercise-related information on short-video social media. (3) I tend to pay attention to fitness- or exercise-related content on short-video social media. (4) For me, short-video social media is an important source of fitness- or exercise-related information. (5) I often watch fitness- or exercise-related content on short-video social media. The five items assess a mixture of self-reported attention, information seeking, and perceived relevance. The measure does not assess objective viewing frequency, duration, or dosage. Items were rated on a 5-point Likert scale from 1 (strongly disagree) to 5 (strongly agree). The mean score was calculated across the 5 item scores and ranged from 1 to 5, with higher scores indicating greater engagement with fitness-related short-video social media content. In the present study, Cronbach’s *α* was 0.94. Confirmatory factor analysis indicated acceptable model fit (χ^2^/*df* = 6.95, RMSEA = 0.10, SRMR = 0.04, CFI = 0.99, TLI = 0.98), and all factor loadings were significant, ranging from 0.77 to 0.94. The CFI and TLI exceeded the conventional 0.95 threshold, indicating excellent relative fit. The RMSEA (0.10), however, was above the commonly recommended cutoff of 0.08. This elevated value, together with the elevated χ^2^/df ratio (6.95), may reflect the model’s small degrees of freedom (*df* = 5), as methodological research has shown that RMSEA can indicate poor fit even for correctly specified models with few degrees of freedom (Kenny et al., 2015; D. Shi et al., 2022). Under such conditions, CFI and SRMR may provide more informative indices of model fit (D. Shi et al., 2022). Given the strong factor loadings (0.77–0.94), excellent CFI (0.99), and acceptable SRMR (0.04), the model was considered acceptable. More importantly, we further conducted multigroup confirmatory factor analysis to examine measurement invariance across gender in the Section 3.2. Configural invariance was supported based on CFI/TLI/SRMR, although RMSEA was elevated (0.092); however, metric invariance was not supported for the engagement scale (Δχ^2^(5) = 54.210, *p* < 0.0001), indicating that factor loadings differed between women and men. Full results are provided in the Section 3.2, Tables S1 and S2. The engagement measure therefore assessed several aspects of engagement with fitness content, including repeated attention, perceived relevance, and informativeness. These aspects of self-reported engagement are broadly consistent with the interpretive framework of cultivation theory, although the present study does not directly assess cultivation media exposure or dosage (Morgan & Shanahan, 2010).

#### 2.2.2. Exercise Identity

The Chinese version of the Exercise Identity Scale was used (Li et al., 2019). It contains 9 items and has a unidimensional structure. All items were rated on a 7-point Likert scale (1 = strongly disagree, 7 = strongly agree). The mean score across all items was calculated, with higher scores indicating stronger identification as an exerciser. Cronbach’s *α* for the total scale was 0.90.

#### 2.2.3. Mindful Eating

The Chinese version of the Mindful Eating Scale was used (Guo et al., 2026). It contains 24 items across six dimensions: awareness, non-reactivity, openness, gratitude, non-judgment, and hunger/satiety. All items were rated on a 5-point Likert scale (1 = never, 5 = always). The mean score across all items was calculated, with higher scores indicating greater mindful eating. Cronbach’s α was 0.88 for the total scale and ranged from 0.83 to 0.89 across the subscales.

#### 2.2.4. Body Appreciation

The Mandarin Chinese version of the Body Appreciation Scale-2 (BAS-2) was used (Swami et al., 2023). It contains 10 items. All items (e.g., “I respect my body”) were rated on a 5-point Likert scale ranging from 1 (never) to 5 (always). The mean score across all items was calculated, with higher scores indicating greater body appreciation. Cronbach’s α for the total scale was 0.93.

### 2.3. Data Analysis

Given that exercise identity and mindful eating may theoretically be associated through both independent and sequential pathways (Anderson & Cychosz, 1994; Pringle et al., 2026; L. Shi et al., 2024; Strachan et al., 2009), we specified a model that included both independent and sequential indirect pathways simultaneously within a multigroup SEM framework. To formally test whether the indirect associations differed by gender, we used Mplus 8.3 (Muthén & Muthén, 2017), and estimated the model simultaneously for women (*n* = 270) and men (*n* = 266), with all structural paths freely estimated across groups. This approach allowed direct statistical comparisons of the indirect effects between women and men using bootstrap confidence intervals (Hayes, 2018; Preacher & Hayes, 2008), rather than relying on separate subgroup analyses.

Age, BMI, and exercise intensity were included as covariates in all analyses. BMI was calculated from self-reported height and weight as weight in kilograms divided by height in meters squared. No implausible values (e.g., height < 140 cm or >200 cm, or weight < 35 kg or >120 kg) were identified in the raw data; therefore, no participants were excluded on this basis. Exercise intensity was assessed using the Physical Activity Rating Scale (PARS-3; Liang, 1994), which measures exercise intensity (“How intense is your physical exercise?”, rated on a 5-point scale from 1 = very light to 5 = very vigorous), duration (“How long do you exercise each time?”, rated on a 5-point scale from 1 = less than 10 min to 5 = more than 60 min), and frequency (“How many times do you exercise per month?”, rated on a 5-point scale from 1 = less than once a month to 5 = approximately once a day). Following the standard PARS-3 scoring procedure, a total physical activity score was computed as (duration − 1) × intensity × frequency (Liang, 1994), with a theoretical range of 0–100. Higher scores indicate greater overall exercise intensity. This composite score was entered as an observed covariate in all analyses. Because the score is a composite of three distinct behavioral dimensions, Cronbach’s α is not an appropriate index of its reliability. Bootstrapping with 5000 resamples was used to construct 95% bias-corrected confidence intervals (CIs) for all indirect effects and group differences (Hayes, 2018). Standardized coefficients (STDYX) are reported. The model was just-identified (χ^2^ = 0, df = 0).

For the ordinal measurement-invariance analyses, WLSMV was used with both delta and theta parameterizations. Under WLSMV, the identification of thresholds, residual variances, and scale parameters depends on the parameterization and the identification constraints imposed on the baseline model (Wu & Estabrook, 2016). Consequently, imposing cross-group equality constraints on thresholds can alter the identification of residual or scale parameters rather than simply increasing the number of equality constraints. In the present analyses, the scalar models therefore had the same number of free parameters as the corresponding metric or partial metric models, and DIFFTEST could not be computed because the models did not form the required nested sequence. Accordingly, scalar invariance was not claimed for any of the measures. Full DIFFTEST results and the threshold constraints are provided in Supplementary Tables S1 and S2.

### 2.4. Clustering Sensitivity Analysis

Because section identifiers were not retained in the final dataset, empirical ICCs could not be estimated, and cluster-adjusted inference could not be conducted. A design-effect sensitivity analysis was therefore conducted to assess the potential impact of within-section dependence on statistical efficiency. Following the standard design-effect approach, the design effect was calculated as DE = 1 + (m − 1)ρ, where *m* represents the average section size and *ρ* represents the ICC (Kish, 1965; Eldridge et al., 2006). The 12 sections had an average size of 44.7 participants. Assuming ICC values ranging from 0.01 to 0.10, the corresponding design effects ranged from 1.44 to 5.37, corresponding to effective sample sizes of approximately 373 to 100, respectively, for the full sample of 536 participants. These estimates illustrate the potential reduction in statistical efficiency under different levels of within-section dependence. However, because the actual ICCs were unavailable, this analysis could not determine the extent to which clustering affected parameter estimates or statistical significance. The sensitivity analysis should therefore be interpreted as an assessment of the potential consequences of clustering, rather than as a substitute for empirical ICC estimation or cluster-adjusted inference.

## 3. Results

### 3.1. Descriptive Statistics and Correlations

Table 1 presents descriptive statistics by gender. Descriptively, women reported higher mean scores than men on engagement, mindful eating, and body appreciation, whereas men reported a higher mean score on exercise identity. As shown in Table 2, engagement was significantly correlated with exercise identity, mindful eating, and body appreciation in both groups. The correlation between exercise identity and mindful eating was significant among women (*r* = 0.35, *p* < 0.001), but not among men (*r* = 0.12, *p* = 0.12). By contrast, exercise identity and mindful eating were each significantly correlated with body appreciation in both groups (all *p* < 0.001).

### 3.2. Measurement Invariance Across Genders

To assess whether the key measures functioned equivalently across genders, we conducted multigroup confirmatory factor analyses (MG-CFAs) for the Fitness-Related Short-video Social Media Engagement Scale, the Exercise Identity Scale, the Mindful Eating Scale, and the Body Appreciation Scale-2 (BAS-2). All items were treated as ordinal variables, and the models were estimated using the weighted least-squares mean- and variance-adjusted estimator (WLSMV) with theta parameterization. Following established guidelines (Chen, 2007; Cheung & Rensvold, 2002), we tested increasingly restrictive models: configural invariance (the same factor structure across groups) and metric invariance (equal factor loadings). Scalar invariance was also evaluated by imposing equality constraints on item thresholds; however, as described in the Section 2.3, the resulting models did not form the required nested sequence for DIFFTEST under the specified identification conditions. Model fit was evaluated using the comparative fit index (CFI), Tucker–Lewis index (TLI), root mean square error of approximation (RMSEA), and standardized root mean square residual (SRMR). Measurement invariance was considered supported when the change in CFI (ΔCFI) between successive models was ≤0.010.

As shown in Table 3, configural invariance was supported for all four scales (CFI ≥ 0.905, SRMR ≤ 0.064). Metric invariance was not supported for the Engagement Scale (Δχ^2^(5) = 54.210, *p* < 0.0001), the Exercise Identity Scale (Δχ^2^(9) = 26.106, *p* = 0.0020), or the Mindful Eating Scale (Δχ^2^(24) = 39.699, *p* = 0.0230). Even after freeing the loadings of E1, E4, and E5, partial metric invariance was not supported for the Engagement Scale (Δχ^2^(2) = 22.087, *p* < 0.0001). Similarly, freeing the loadings of M7, M19, and M22 did not achieve partial metric invariance for the Mindful Eating Scale (Δχ^2^(21) = 39.262, *p* = 0.0091). For the Body Appreciation Scale, metric invariance was not supported (Δχ^2^(10) = 27.241, *p* = 0.0024). However, after freeing the factor variance in the female group, partial metric invariance was supported (Δχ^2^(9) = 2.944, *p* = 0.9665), suggesting that the factor loadings could be treated as invariant across genders under this identification condition, whereas the estimated factor variance was lower among women than among men (0.638 vs. 1.000). Scalar invariance could not be reliably tested for any of the scales. Under WLSMV, the identification of thresholds and residual or scale parameters depends on the parameterization and the identification constraints. Consequently, cross-group equality constraints on thresholds may not increase the degrees of freedom in the expected manner. As a result, the scalar models had the same number of free parameters as the metric or partial metric models, and DIFFTEST could not be computed. Accordingly, scalar invariance was not claimed for any of the measures. Full DIFFTEST results and threshold constraints are provided in Supplementary Tables S1 and S2.

Although RMSEA values for some models exceeded the conventional 0.08 cutoff, elevated RMSEA values are well documented in models with few degrees of freedom (Kenny et al., 2015; D. Shi et al., 2022). Under such conditions, CFI and SRMR may provide more reliable information about model fit (Kenny et al., 2015). For the Mindful Eating Scale, configural invariance was supported (CFI = 0.905, TLI = 0.904, RMSEA = 0.083, SRMR = 0.063), whereas metric invariance was not supported (Δχ^2^(24) = 39.699, *p* = 0.0230), and partial metric invariance was also not supported (Δχ^2^(21) = 39.262, *p* = 0.0091). Scalar invariance could not be reliably tested. Given the lack of support for metric invariance, the six facets were not compared across genders. Instead, consistent with the study’s focus on global mindful eating, the total score was used as an observed composite in the structural model. This approach preserved model parsimony but may obscure potential facet-level differences, which should be examined in future research. Taken together, the CFI, TLI, SRMR, and available invariance evidence provided some support for cross-gender comparability, although this support was not uniform across measures. For the Exercise Identity Scale, however, the invariance evidence should be interpreted cautiously because the configural model showed suboptimal absolute fit. Accordingly, subsequent comparisons of structural paths and indirect effects should be interpreted in light of these measurement limitations.

### 3.3. Multigroup Structural Equation Modeling Analysis

As noted above, metric invariance was not supported for the Engagement, Exercise Identity, or Mindful Eating scales. Accordingly, structural path estimates involving these variables and between-gender differences in indirect effects should be interpreted with caution, as these comparisons may reflect differences in the measurement properties of the constructs as well as differences in regression slopes. The model was estimated simultaneously for women (n = 270) and men (n = 266) groups, with all structural paths freely estimated across groups. This approach allowed direct statistical comparisons of indirect effects between women and men using bootstrap confidence intervals. Age, BMI, and exercise intensity were included as covariates in all regression equations. Bootstrapping with 5000 resamples was used to construct 95% bias-corrected confidence intervals (CIs) for all indirect effects and group differences. Table 4 reports STDYX-standardized path coefficients, whereas Table 5 reports unstandardized indirect effects and between-gender contrasts derived from the MODEL CONSTRAINT procedure, with statistical significance evaluated using the corresponding bootstrap confidence intervals. The model was just-identified (χ^2^ = 0, df = 0). Because the model was just-identified, global fit indices such as CFI, TLI, RMSEA, and SRMR were not informative and were therefore not interpreted. Results are summarized in Table 4 and Table 5.

As shown in Table 4, the associations among the study variables varied across genders. Among men, exercise identity was significantly associated with body appreciation (*β* = 0.327, *p* < 0.001), as was mindful eating (*β* = 0.446, *p* < 0.001). Among women, mindful eating was significantly associated with body appreciation (*β* = 0.447, *p* < 0.001), whereas the association between exercise identity and body appreciation was not significant (*β* = 0.030, *p* = 0.695). Engagement was significantly associated with exercise identity among both men (*β* = 0.474, *p* < 0.001) and women (*β* = 0.458, *p* < 0.001). Exercise identity was significantly associated with mindful eating only among women (*β* = 0.359, *p* < 0.001), but not among men (*β* = 0.045, *p* = 0.569).

The indirect effects and between-gender contrasts are presented in Table 5. The serial indirect association from engagement through exercise identity and mindful eating to body appreciation was significant among women (effect = 0.069, 95% CI [0.037, 0.111]), but not among men, and the between-gender difference was statistically significant (diff = 0.062, 95% CI [0.019, 0.111]). In contrast, the indirect association through exercise identity alone (Engagement → Exercise identity → Body appreciation) was significant only among men (effect = 0.124, 95% CI [0.059, 0.199]), but not among women, with a significant between-gender difference (diff = −0.111, 95% CI [−0.204, −0.022]). The indirect association through mindful eating alone (Engagement → Mindful eating → Body appreciation) was not significant in either group, and the between-gender difference was also not statistically significant (diff = −0.078, 95% CI [−0.163, 0.002]). The total indirect effects was significant among men (effect = 0.186, 95% CI [0.099, 0.279]), but not among women (effect = 0.058, 95% CI [−0.016, 0.140],see Table 5). Total effects were also significant among both women (effect = 0.169, 95% CI [0.053, 0.287]) and men (effect = 0.238, 95% CI [0.117, 0.360]). By contrast, direct effects were not significant in either group (women: effect = 0.117, 95% CI [−0.013, 0.227]; men: effect = 0.065, 95% CI [−0.049, 0.163]).

Overall, the results indicated a gender-differentiated pattern of indirect associations. Among women, the significant indirect association followed the serial pathway Engagement → Exercise identity → Mindful eating → Body appreciation. Among men, the significant indirect association was observed through exercise identity, whereas the indirect association through mindful eating was not significant. Formal between-gender comparisons indicated significant differences in the serial indirect association and in the indirect association through exercise identity.

**Figure 1:**
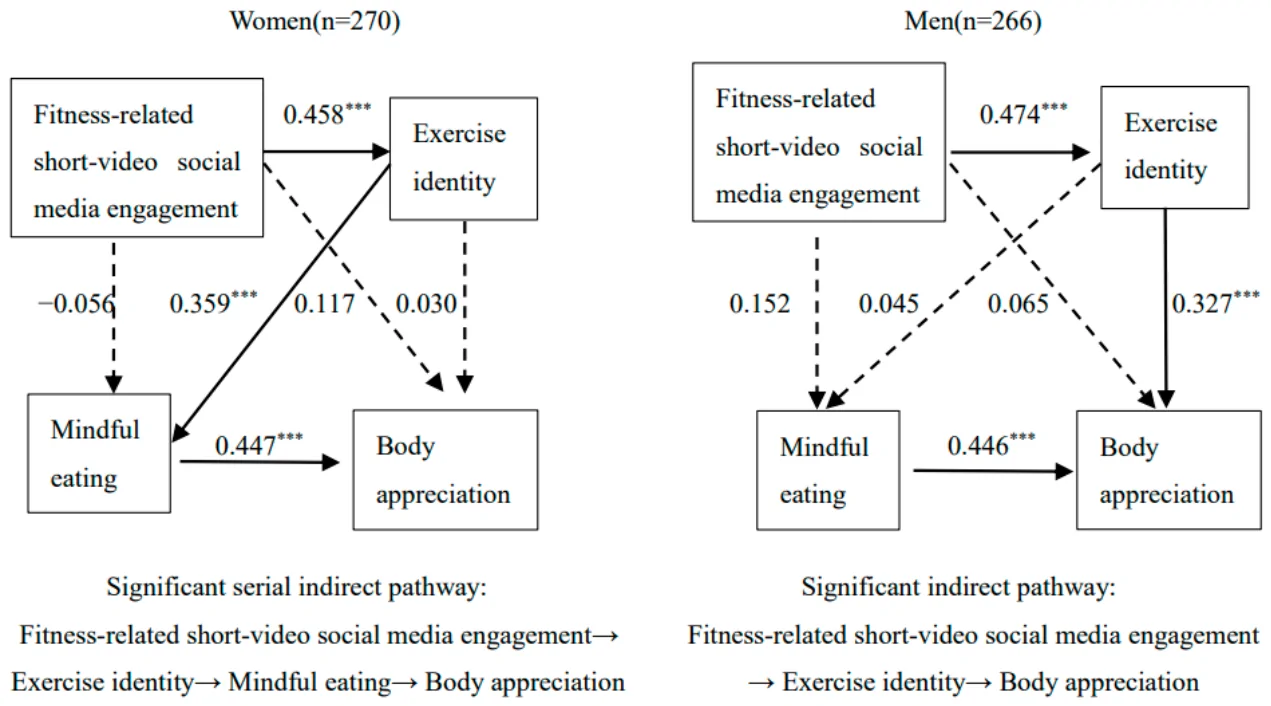
Gender-differentiated multigroup indirect pathway model for body appreciation. Path coefficients are from the main model with exercise intensity included as a covariate. A sensitivity analysis without exercise intensity is reported in Table 6. *** *p* < 0.001.

Regarding the covariates, BMI was significantly positively associated with body appreciation among women (*β* = 0.156, *p* = 0.002), whereas age was significantly negatively associated with body appreciation among men (*β* = −0.113, *p* = 0.029). Exercise intensity was significantly associated with exercise identity in both groups (men: *β* = 0.356, *p* < 0.001; women: *β* = 0.311, *p* < 0.001). Engagement was also significantly associated with exercise identity in both groups. Figure 1 displays the standardized path coefficients for women and men, illustrating the gender-differentiated pattern of associations.

**Table 6:** Sensitivity analysis: Indirect effects with and without exercise intensity as a covariate.

Pathway	With Exercise Intensity Effect [95% CI]	Without Exercise Intensity Effect [95% CI]
Women		
Engagement →Exercise identity →Body appreciation	0.013 [−0.043, 0.076]	0.152 [0.082, 0.228]
Engagement → Mindful eating→ Body appreciation	−0.024 [−0.083, 0.033]	—
Engagement →Exercise identity → Mindful eating → Body appreciation	0.069 [0.037, 0.111]	0.006 [−0.026, 0.036]
Men		
Engagement →Exercise identity →Body appreciation	0.124 [0.059, 0.199]	0.037 [−0.024, 0.108]
Engagement → Mindful eating→ Body appreciation	0.054 [−0.002, 0.114]	—
Engagement →Exercise identity → Mindful eating → Body appreciation	0.008 [−0.019, 0.034]	0.087 [0.051, 0.140]
Gender differences (W–M)		
Δ (Exercise identity →Body appreciation)	−0.111 [−0.204, −0.022]	−0.115 [−0.214, −0.019]
Δ (Serial)	0.062 [0.019, 0.111]	0.081 [0.031, 0.140]

Note. All effects are STDYX-standardized estimates with 5000 bootstrap resamples. The model with exercise intensity includes age, BMI, and exercise intensity as covariates. The model without exercise intensity includes only age and BMI.

### 3.4. Sensitivity Analysis: With and Without Exercise Intensity

To examine whether adjustment for exercise intensity influenced the estimated indirect effects, we re-estimated the multigroup model without exercise intensity as a covariate, while retaining age and BMI. As shown in Table 6, the pattern of statistically significant indirect effects differed between the two model specifications. In the model with exercise intensity, the indirect association through exercise identity was significant only among men (0.124, 95% CI [0.059, 0.199]), whereas the serial indirect association was significant only among women (0.069, 95% CI [0.037, 0.111]). In the model without exercise intensity, the pattern of statistical significance differed: the indirect association through exercise identity was significant only among women (0.152, 95% CI [0.082, 0.228]), whereas the serial indirect association was significant only among men (0.087, 95% CI [0.051, 0.140]). The between-gender differences in both indirect associations remained statistically significant and in the same respective directions across model specifications (Δ identity: −0.115, 95% CI [−0.214, −0.019]; Δ serial: 0.081, 95% CI [0.031, 0.140]). These findings suggest that the direction of the between-gender differences was relatively stable across specifications, whereas the gender-specific statistical significance of the individual indirect associations depended on whether exercise intensity was included as a covariate. The path coefficients presented in Figure 1 are from the main model with exercise intensity included as a covariate.

## 4. Discussion

As noted above, metric invariance was not supported for the Engagement, Exercise Identity, or Mindful Eating scales. Therefore, the gender-differentiated patterns reported here should be interpreted cautiously, because they may reflect differences in measurement properties as well as differences in regression slopes. Accordingly, these findings should not be interpreted as evidence of gender differences in latent means or latent structural relationships. Future research using measures that demonstrate stronger and more consistent measurement invariance is needed to determine whether the observed gender-differentiated patterns are replicated when measurement properties are more comparable across genders.

The present study examined whether exercise identity and mindful eating accounted for indirect associations between engagement and body appreciation among Chinese college students, with particular attention to whether these indirect associations differed by gender. The findings indicated that the pattern of indirect associations between engagement and body appreciation differed between women and men. Formal between-gender comparisons supported this interpretation, revealing significant differences in both the serial indirect association (Women-Men = 0.062, 95% CI [0.019, 0.111]), indicating that the serial indirect effect was significantly larger among women than among men, and the indirect association through exercise identity (diff = −0.111, 95% CI [−0.204, −0.022]), indicating that this indirect effect was significantly larger among men than among women. The total effects of engagement on body appreciation were significant in both women (effect = 0.169, 95% CI [0.053, 0.287]) and men (effect = 0.238, 95% CI [0.117, 0.360]), whereas the direct effects were not significant in either group (women: effect = 0.117, 95% CI [−0.013, 0.227]; men: effect = 0.065, 95% CI [−0.049, 0.163]). Among women, the total indirect effect was not significant, although the serial indirect association was significant. Among men, the total indirect effect was significant, with the indirect association through exercise identity accounting for a significant component of this association. Because the study was cross-sectional, these findings are interpreted as patterns of statistical indirect association rather than evidence of temporal or causal mediation.

The positive association between BMI and body appreciation among women is noteworthy. One speculative possibility, not tested in the present study, is that higher BMI may reflect greater body acceptance among women who are exposed to body-positive or health-focused fitness content, consistent with perspectives emphasizing body functionality and self-acceptance (Alleva & Tylka, 2021; Tylka & Wood-Barcalow, 2015). However, because the present study did not distinguish body-positive from appearance-focused fitness content, this explanation remains speculative and should be examined in future research. Alternatively, this association may be cohort-specific, reflecting shifting cultural norms around body size and beauty standards among contemporary Chinese female college students. Given the restricted age range (18–26 years), the negative association between age and body appreciation among men should be interpreted tentatively. One possible explanation is that body appreciation may decline as men progress through college and experience greater social comparison related to muscularity and physical competence (Cash & Smolak, 2011; Voges et al., 2019). However, these explanations were not directly tested in the present study. Future longitudinal research is needed to clarify these age-related patterns and the factors that may account for them.

A central finding was the contrast between the serial indirect pattern observed among women and the more direct identity-based indirect pattern observed among men. For young women, the significant indirect association between engagement and body appreciation followed the sequential chain of exercise identity → mindful eating → body appreciation. One possible interpretation is that this pattern is consistent with a process in which engagement is associated with stronger exercise identity, which is in turn associated with more attentive and nonjudgmental eating-related cognition and, subsequently, greater body appreciation. However, because the present study was cross-sectional, the temporal ordering implied by this sequence cannot be established. Future longitudinal research should examine whether these associations unfold in this order over time. Prior research suggests that women’s body evaluations may be more closely linked to weight control, dietary monitoring, and eating-related self-regulation (Fredrickson & Roberts, 1997; Tiggemann, 2004). In this context, exercise participation or exercise identity may not be sufficient, on its own, to be associated with more positive body attitudes; instead, mindful eating may provide an additional form of behavioral-cognitive integration (L. Shi et al., 2024; Strachan & Brawley, 2008). The absence of significant independent indirect pathways through exercise identity or mindful eating alone among women is consistent with the possibility that these constructs are associated jointly rather than as fully separable cognitive resources. One possible interpretation, drawing on a self-objectification perspective, is that girls and women may be socialized to evaluate bodily worth partly through appearance and eating restraint across adolescence and young adulthood (Fredrickson & Roberts, 1997; Moradi & Huang, 2008). Although objectification and dietary restraint were not directly measured in the present study, this perspective may help contextualize why an exercise identity may not independently relate to body appreciation among women. Future research should test whether these sociocultural mechanisms explain the observed gender differences. This dynamic may be especially salient within the Chinese cultural context, where peer and family discourse can reinforce norms surrounding dietary restraint and body shape. Although exercise identity is generally considered adaptive, a rigid or appearance-driven exercise identity may also be associated with compulsive exercise or disordered eating (Palermo & Rancourt, 2021). Accordingly, the observed serial indirect association may depend on the form or quality of exercise identity rather than on exercise identity alone. Future research could distinguish between autonomous and controlled forms of exercise motivation and examine whether motivation type is associated with variation in the serial indirect association.

Most previous research on media and body image has focused on other mediating processes, such as social comparison and self-objectification, with comparatively less attention to exercise identity, mindful eating, or their sequential interplay. The present findings contribute to this literature by identifying a significant serial indirect pathway among Chinese college women, thereby highlighting the potential interrelatedness of exercise identity- and eating-related awareness in the association between fitness-related media engagement and body appreciation. Among men, by contrast, the significant indirect effect operated through exercise identity alone. Mindful eating was strongly associated with body appreciation, but engagement was not significantly associated with mindful eating, and the corresponding indirect effect was not statistically significant. This null finding should nevertheless be interpreted cautiously, as a small indirect association cannot be ruled out on the basis of the confidence interval alone. This pattern may tentatively suggest that exercise self-concept and eating-related awareness may be less closely connected in the association between fitness-related media engagement and men’s body appreciation, although this interpretation should be treated with caution given that the pathway was not statistically significant and the confidence interval was wide. Consistent with this pattern, the association between exercise identity and mindful eating was not statistically significant among men. One possible interpretation, drawing on prior research documenting gender differences in body-value orientations (Cash & Smolak, 2011; Voges et al., 2019), is that men’s body image concerns may place greater emphasis on physical functionality, muscularity, and athletic competence than on dietary control. However, because muscularity pressure and cognitive compartmentalization were not directly measured in the present study, future research should test whether these factors help account for the observed gender differences. More broadly, the findings illustrate why models that pool women and men may obscure potential gender differences in the associations linking fitness-related media engagement with positive body image. The pattern observed among men complements the serial indirect association observed among women and provides preliminary evidence of gender-differentiated indirect associations in the context of fitness-related media engagement. However, because the indirect association through mindful eating was not significant among men, future longitudinal research could examine whether this association varies across different forms or levels of engagement.

The sensitivity analysis with and without exercise intensity revealed that the specific group in which each indirect pathway reached significance depended on whether exercise intensity was included. This finding indicates that the estimated indirect associations were sensitive to adjustment for exercise intensity. However, the sensitivity analysis alone does not establish whether exercise intensity functions as a confounder, a mediator, or another variable related to the observed associations. When exercise intensity is statistically controlled, the estimated associations among engagement, exercise identity, and body appreciation may change because variance shared with exercise intensity is accounted for, potentially altering the magnitude and statistical significance of the indirect associations across groups. These findings underscore the conceptual complexity of adjusting for exercise intensity in models of media engagement and body image, and suggest that future research should use longitudinal or experimental designs to clarify the temporal and conceptual role of exercise behavior in these associations.

These findings should nevertheless be interpreted cautiously because the data are correlational and cross-sectional. Although the observed patterns are consistent with the hypothesized gender-differentiated indirect associations, they do not establish causal or temporal relationships. The results are therefore best viewed as preliminary, hypothesis-generating evidence rather than evidence of causal mechanisms.

## 5. Implications

These findings are broadly consistent with a cultivation-theory perspective in suggesting that self-reported engagement with fitness-related short-video content is associated with self-relevant identities and behavioral orientations. The observed patterns differed by gender: among women, the significant indirect association followed a sequential pattern involving exercise identity, mindful eating, and body appreciation; among men, the significant indirect association was observed through exercise identity. Practically, the findings caution against one-size-fits-all approaches to health and body image interventions. For women, future intervention research could examine whether approaches that integrate exercise-identity development with body-aware eating practices are associated with more favorable body-image outcomes than approaches focused on exercise identity alone. For men, intervention studies could separately examine the potential relevance of exercise identity and mindful eating to body appreciation. These implications remain tentative, particularly given the cross-sectional design and measurement limitations, and should be evaluated in experimental or longitudinal intervention studies before being translated into practice.

## 6. Conclusions

The present study provides preliminary evidence that self-reported engagement with fitness-related short-video content is associated with body appreciation through gender-differentiated indirect pathways. Among women, the association followed a serial pathway through exercise identity and mindful eating. Among men, it operated through exercise identity, whereas the indirect pathway through mindful eating was not significant. However, metric invariance was not supported for the engagement, exercise identity, and mindful eating scales, and scalar invariance could not be reliably tested for any measure. Given the cross-sectional design, the findings are best viewed as hypothesis-generating and should be replicated in longitudinal and experimental research.

### Limitations and Future Directions

Despite its strengths, this study has several limitations. First, the cross-sectional design precludes causal inference. Longitudinal and experimental studies are needed to establish temporal precedence. Second, the sample was limited to Chinese college students aged 18–26 years recruited from three universities. Because section identifiers were not retained, section-level ICCs could not be estimated and cluster-robust inference could not be conducted. Future studies should retain section- or session-level identifiers and consider multilevel approaches. Third, all variables were assessed using self-report measures, which may be susceptible to social-desirability bias, recall bias, and common-method variance. Future studies could integrate behavioral measures (e.g., objective exercise tracking and dietary records) and digital indicators of media exposure (e.g., viewing time, frequency, algorithmic recommendation patterns). Fourth, measurement invariance was limited. Metric invariance was not supported for the engagement, exercise identity, or mindful eating scales, and scalar invariance could not be reliably tested. Only the body appreciation scale achieved partial metric invariance. Gender comparisons involving the non-invariant latent variables should therefore be interpreted cautiously, because observed group differences may reflect differences in measurement properties as well as differences in regression slopes. Future research should examine alternative structures using ESEM or alignment optimization. The adapted engagement scale showed an elevated RMSEA (0.10), possibly due to local dependence among its five conceptually overlapping items. Since it was adapted from a fashion measure and validated only by expert review and a small pilot test, its construct validity in the fitness context remains provisional and should be examined in future research. Fifth, the present study focused on exercise identity and mindful eating as mediating processes, but other factors—such as appearance comparison, self-compassion, and appreciation of body functionality—may also contribute. Different forms of fitness content (e.g., instructional, inspirational, or appearance-focused content) may also have distinct psychological correlates. Moreover, direct empirical evidence linking exercise identity specifically to mindful eating remains limited, and this pathway was therefore tested as a theoretically plausible but relatively underexamined hypothesis rather than an established relationship. Sixth, the study did not fully address the potentially bidirectional relationship between engagement and exercise-related behavior. Although exercise intensity was statistically controlled, individuals with stronger exercise identities may actively seek fitness-related content. Moreover, the sensitivity analysis with and without exercise intensity showed that the specific group in which each indirect pathway reached significance depended on whether exercise intensity was included, indicating that the estimated indirect associations were sensitive to adjustment for exercise intensity. This sensitivity analysis alone does not establish whether exercise intensity functions as a confounder, a mediator, or another variable related to the observed associations. The direction and statistical significance of specific indirect associations therefore varied across model specifications, although the between-gender differences in these indirect associations remained statistically significant. Future research should carefully consider the conceptual and temporal role of exercise behavior in these associations. Finally, gender was assessed using a binary woman–man item; future studies should include more diverse gender identities.

## Figures and Tables

**Table 1:** Descriptive statistics for the study variables by gender.

Variables	Min	Max	*M* ± *SD*
Engagement	1/1	5/5	3.35 ± 0.82/3.05 ± 1.10
Exercise identity	1/1	7/7	3.96 ± 1.18/4.12 ± 1.36
Mindful eating	2.13/2.13	4.25/4.08	3.07 ± 0.37/3.04 ± 0.35
Body appreciation	1.5/1	5/5	3.97 ± 0.77/3.82 ± 0.88

Note. N = 536 (270 women and 266 men). Values to the left of the slash are for women; values to the right are for men.

**Table 2:** Correlations among the study variables.

	1	2	3	4
Engagement	1			
Exercise identity	0.54 ***/0.59 ***	1		
Mindful eating	0.15 */0.18 **	0.35 ***/0.12	1	
Body appreciation	0.22 ***/0.33 ***	0.30 ***/0.41 ***	0.48 ***/0.49 ***	1

Note. * *p* < 0.05; ** *p* < 0.01; *** *p* < 0.001.

**Table 3:** Measurement invariance tests for the study scales.

Model	χ^2^	*df*	CFI	TLI	RMSEA	90% CI	SRMR	ΔCFI
Fitness-related short-video social media engagement								
Configural	81.231	25	0.997	0.998	0.092	[0.070, 0.114]	0.023	—
Metric	148.957	30	0.995	0.996	0.122	[0.103, 0.141]	0.039	−0.002
Partial Metric	103.806	27	0.997	0.997	0.103	[0.083, 0.124]	0.029	0.000
Scalar	—	—	—	—	—	—	—	—
Exercise identity								
Configural	380.816	99	0.976	0.982	0.103	[0.092, 0.114]	0.052	—
Metric	365.887	108	0.978	0.985	0.094	[0.084, 0.105]	0.055	+0.002
Scalar	—	—	—	—	—	—	—	—
Mindful Eating								
Configural	1542.106	546	0.905	0.904	0.083	[0.078, 0.087]	0.063	—
Metric	1470.817	570	0.914	0.917	0.077	[0.072, 0.082]	0.064	+0.009
Scalar	—	—	—	—	—	—	—	—
Body appreciation								
Configural	326.698	100	0.989	0.990	0.092	[0.081, 0.103]	0.040	—
Metric	315.714	110	0.990	0.992	0.084	[0.073, 0.094]	0.039	+0.001
Partial Metric	324.522	109	0.989	0.991	0.086	[0.075, 0.097]	0.040	0.000
Scalar	—	—	—	—	—	—	—	—

Note. All measurement invariance models were estimated using weighted least-squares mean- and variance-adjusted estimator (WLSMV) with THETA parameterization. Metric invariance was not supported for the Engagement, Exercise Identity, or Mindful Eating Scales. Partial metric invariance was supported only for the Body Appreciation Scale after freeing the female group’s factor variance (Δχ^2^(9) = 2.944, *p* = 0.9665). Scalar invariance could not be reliably tested for any scale because, under WLSMV, cross-group threshold equality constraints were absorbed into residual variances without increasing the number of free parameters. ΔCFI represents the change relative to the preceding model (metric vs. configural; scalar vs. metric). CFI = comparative fit index; TLI = Tucker–Lewis index; RMSEA = root mean square error of approximation; SRMR = standardized root mean square residual.

**Table 4:** Standardized path coefficients from the multigroup SEM by gender.

Path	Women (n = 270) β	Men (n = 266) β
Outcome: Body appreciation		
Identity → Body appreciation	0.030	0.327 ***
Mindful eating → Body appreciation	0.447 ***	0.446 ***
Engagement → Body appreciation (direct)	0.117	0.065
Age → Body appreciation	−0.053	−0.113 *
BMI → Body appreciation	0.156 **	−0.012
Exercise intensity → Body appreciation	0.107	−0.013
Outcome: Exercise identity		
Engagement → Exercise identity	0.458 ***	0.474 ***
Age → Exercise identity	−0.109 *	−0.013
BMI → Exercise identity	0.001	0.030
Exercise intensity → Exercise identity	0.311 ***	0.356 ***
Outcome: Mindful eating		
Engagement → Mindful eating	−0.056	0.152
Identity → Mindful eating	0.359 ***	0.045
Age → Mindful eating	−0.016	0.112 *
BMI → Mindful eating	−0.089	−0.241 ***
Exercise intensity → Mindful eating	0.043	−0.032
R^2^		
Exercise identity	0.393	0.460
Mindful eating	0.134	0.097
Body appreciation	0.299	0.378

Note. The path analysis was estimated using maximum likelihood (ML) with 5000 bootstrap samples. *β* = standardized coefficient. * *p* < 0.05; ** *p* < 0.01; *** *p* < 0.001. Because metric invariance was not supported for the engagement, exercise identity, and mindful eating scales, these estimates should be interpreted with caution, and gender differences reflect differences in regression slopes rather than differences in latent means.

**Table 5:** Indirect effects, direct effects, total effects and gender differences with bootstrap confidence intervals.

Pathway	Women Effect [95% CI]	Men Effect [95% CI]
Specific indirect effects		
Engagement → Exercise identity → Body appreciation (specific IE)	0.013 [−0.043, 0.076]	0.124 [0.059, 0.199]
Engagement → Mindful eating → Body appreciation (specific IE)	−0.024 [−0.083, 0.033]	0.054 [−0.002, 0.114]
Engagement → Exercise identity → Mindful eating → Body appreciation (specific IE)	0.069 [0.037, 0.111]	0.008 [−0.019, 0.034]
Total indirect effect	0.058 [−0.016, 0.140]	0.186 [0.099, 0.279]
Direct effect	0.117 [−0.013, 0.227]	0.065 [−0.049, 0.163]
Total effect	0.169 [0.053, 0.287]	0.238 [0.117, 0.360]
Gender differences (Women–Men)		
Δ (Engagement → Exercise identity → Body appreciation)	−0.111 [−0.204, −0.022]	
Δ (Engagement → Mindful eating → Body appreciation)	−0.078 [−0.163, 0.002]	
Δ (Engagement → Exercise identity → Mindful eating → Body appreciation)	0.062 [0.019, 0.111]	

Note. All models were estimated using ML with 5000 bootstrap samples. CI = confidence interval based on 5000 bootstrap resamples.

## Data Availability

The datasets generated by the survey research during and analyzed during the current study are available in the ScienceDB repository: https://doi.org/10.57760/sciencedb.43569.

## Supplementary Materials

The following supporting information can be downloaded at https://www.mdpi.com/article/10.3390/bs16091685/s1. File S1: Ethical approval; Table S1: Full DIFFTEST results for measurement invariance models; Table S2: Threshold constraints for scalar invariance models.
