# Supplementary material for: Fitness-Related Short Video Engagement and Body Appreciation: Gender-Differentiated Indirect Pathways via Exercise Identity and Mindful Eating
Source: Behav Sci (Basel). 2026 Sep 18;16(9):1685. doi: 10.3390/bs16091685 (PMC13603422; doi:10.3390/bs16091685)
Supplement: Supplementary file 1 [file behavsci-16-01685-s001.zip › behavsci-4501839-supplementary.pdf]

Supplementary File S1 Ethical Approval

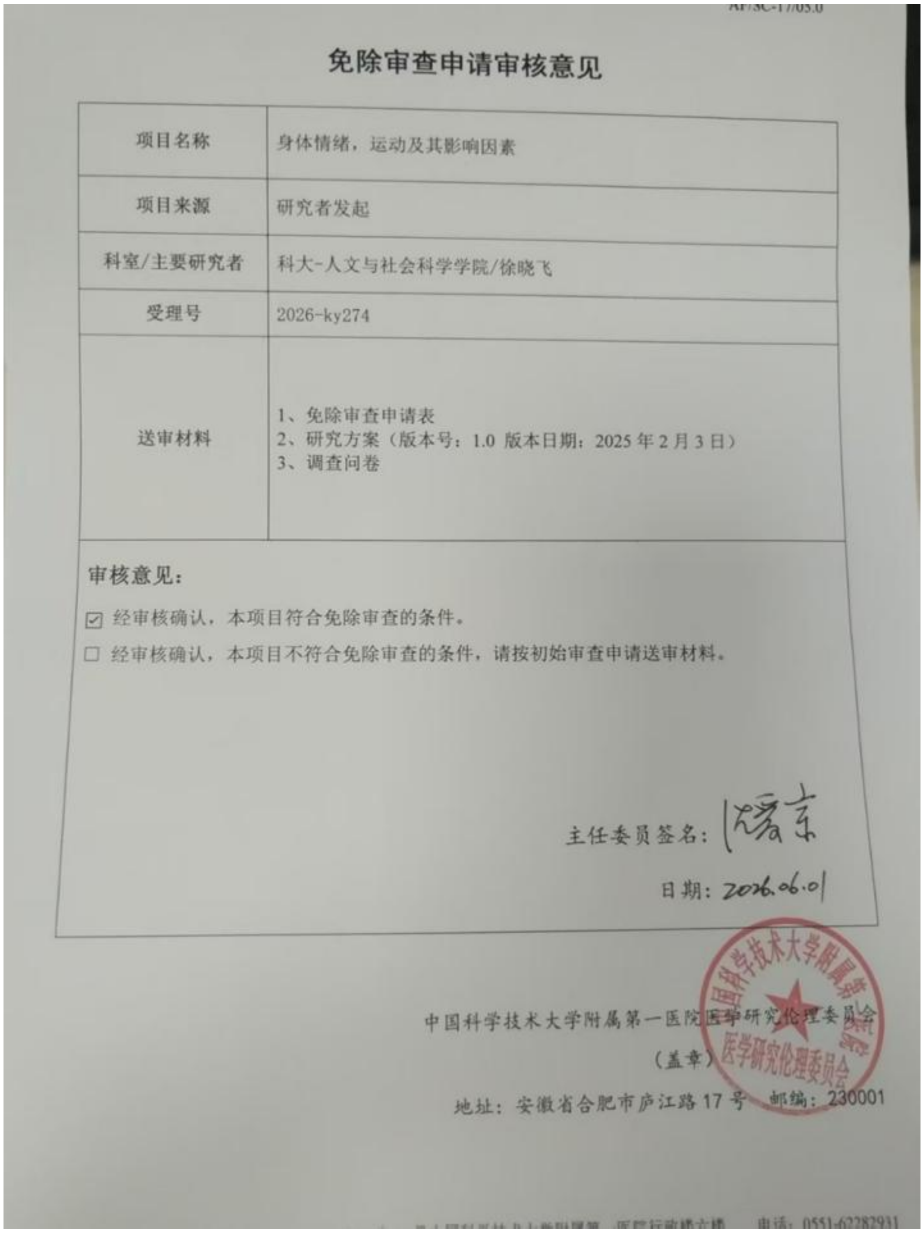

AF/SC-17/05.0

免除审查申请审核意见

项目名称	身体情绪，运动及其影响因素
项目来源	研究者发起
科室/主要研究者	科大-人文与社会科学学院/徐晓飞
受理号	2026-ky274
送审材料	1、免除审查申请表<br>2、研究方案（版本号：1.0 版本日期：2025 年 2 月 3 日）<br>3、调查问卷

审核意见：

☑ 经审核确认，本项目符合免除审查的条件。

☐ 经审核确认，本项目不符合免除审查的条件，请按初始审查申请送审材料。

主任委员签名：[illegible]

日期：2026.06.01

中国科学技术大学附属第一医院医学研究伦理委员会

（盖章）

地址：安徽省合肥市庐江路 17 号　邮编：230001

[illegible]

Exemption Review Application Review Opinion

Item	Content
**Project Title**	Body Emotion, Exercise, and Their Influencing Factors
**Project Source**	Investigator-Initiated
**Department / Principal Investigator**	USTC – School of Humanities and Social Sciences / Xu Xiaofei
**IRB Number**	2026-ky274
**Submitted Materials**	1. Exemption Review Application Form<br>2. Research Protocol (Version 1.0 / Date: February 3, 2025)<br>3. Questionnaire

Supplementary file S2

Table S1. Full DIFFTEST results for measurement invariance models.

Scale	Comparison	$\Delta\chi^2$	df	p
Engagement	Metric vs Configural	54.210	5	<0.0001
Engagement	Partial Metric vs Configural	22.087	2	<0.0001
Engagement	Scalar vs Metric	—	—	not computable
Exercise Identity	Metric vs Configural	26.106	9	0.0020
Exercise Identity	Scalar vs Metric	—	—	not computable
Mindful Eating	Metric vs Configural	39.699	24	0.0230
Mindful Eating	Partial Metric vs Configural	39.262	21	0.0091
Mindful Eating	Scalar vs Metric	—	—	not computable
Body Appreciation	Metric vs Configural	27.241	10	0.0024
Body Appreciation	Partial Metric vs Configural	2.944	9	0.9665
Body Appreciation	Scalar vs Partial Metric	—	—	not computable

Note. DIFFTEST was used to compare nested models. Scalar comparisons are marked “not computable” because the scalar models had the same number of free parameters as the metric or partial metric models, and the models were not nested in terms of parameter count.

Supplementary Table S2. Threshold constraints for scalar invariance models.

Scale	Item	Thresholds constrained equal	Thresholds fixed for identification
Engagement	E1–E5	$2, $3, $4	$1
Exercise Identity	I1–I9	$2–$6	$1
Mindful Eating	M1–M24	$2–$4	$1
Body Appreciation	B1–B10	$2–$4	$1

Note. Under WLSMV, the first threshold of each item was fixed for identification. The remaining thresholds were constrained equal across groups in the scalar models. Because the thresholds and residual variances were not separately identified under WLSMV, these constraints were absorbed into the residual variances and did not increase the number of free parameters.
